# International Commission for the Restoration of Hahnemann’s Tomb

**Published:** 1898-04

**Authors:** Bushrod W. James


### INTERNATIONAL COMMISSION FOR THE RESTORATION OF HAHNEMANN'S TOMB.

Very few of the followers of Homœopathy are aware of the sad fact that the grave of Samuel Hahnemann, the founder of the homœopathic method of treatment, in the cemetery of Montmartre in Paris, is in a very greatly neglected condition, the body having lain there for fully fifty years and the surroundings having gradually and almost completely decayed.

The Quinquennial International Congress of 1896, held in London, which coincided with the year of the celebration of the centenary of Homœopathy, resolved to signalize this event by the restoration of Hahnemann's tomb; and in order to carry this resolution into effect elected an Internationl Executive Commission, composed of the members whose names are signed below.

It was the duty of the Commission, first of all, to secure the consent of the owners of the grave to the carrying out of the necessary works and to the legal transfer of it in perpetuity to the French Homœopathic Society, to be maintained by that body.

This task has been fulfilled.

The Commission will now have to occupy itself with the financial side of the matter, and with this object it has opened an international subscription, and now appeals to all homœopathic societies, to all homœopathic physicians, and to all followers of Homœopathy throughout the whole world with an earnest request for assistance.

It is impossible to longer suffer that the grave which preserves the mortal remains of one of the greatest physicians and benefactors of mankind should remain in such lamentable neglect, and the Commission hopes that every one enjoying the inestimable benefits of homœopathic treatment will consider it a matter of honor to contribute his mite towards the erection of a monument worthy of the undying fame of Samuel Hahnemann.

Subscriptions are received by the members of the Commission, or are sent straight to the Secretary of the Commission in Paris. The list of subscriptions will be printed in the *Revue Homœopathique Francaise* and other journals of the countries represented on the Commission.

LEON BRASOL, M. D., *Chairman*, Russia.
St. Petersburg, Nikolaievskaia, 8.

FRANCOIS CARTIER, M. D., *Secretary*, France.
Paris, 18 Rue Vignon.

RICHARD HUGHES, M. D., England.
Brighton, 36 Sillwood Road.

BUSHROD W. JAMES, M. D., U. S. America.
Philadelphia, Pa., N. E. Cor. Eighteenth and Green Streets.

ALEXANDER VILLERS, M. D., Germany.
Dresden, Luttichaustrasse 7.

---

TO THE MEMBERS OF OUR MEDICAL PROFESSION:

In accordance with the desire of the International Commission to improve Hahnemann's tomb, the above appeal is sent you for consideration and action. As the American representative I will be glad to hear from you.

Very truly and fraternally yours,

BUSHROD W. JAMES.

PHILADELPHIA, PA., April 1st, 1898.

